# Effects of Water Stress, Defoliation and Crop Thinning on *Vitis vinifera* L. cv. Solaris: Part I: Plant Responses, Fruit Development and Fruit Quality

**DOI:** 10.3390/metabo12040363

**Published:** 2022-04-18

**Authors:** Violetta Aru, Andreas Paul Nittnaus, Klavs Martin Sørensen, Søren Balling Engelsen, Torben Bo Toldam-Andersen

**Affiliations:** 1 Department of Food Science, University of Copenhagen, Rolighedsvej 26, DK-1958 Frederiksberg, Denmark; violetta@food.ku.dk (V.A.); kms@food.ku.dk (K.M.S.); se@food.ku.dk (S.B.E.); 2 Department of Plant and Environmental Sciences, University of Copenhagen, Højbakkegård Alle 13, DK-2630 Taastrup, Denmark; a.nittnaus@gmx.at; 3 Instituto Superior de Agronomia, Universidade de Lisboa, Tapada da Ajuda, 1349-017 Lisboa, Portugal

**Keywords:** *Vitis vinifera*, Solaris, grapevine, climate change, water stress, defoliation, crop-thinning, FT-IR, bulk grape metabolome

## Abstract

Viticultural practices and irrigation have a major impact on fruit development and yield, and ultimately on must quality. The effects of water deficit (WD), defoliation (Def), and crop-thinning (CT) on Solaris plants and fruit development, as well as on the chemical composition of grape juice were investigated. WD was induced at three periods during fruit development (pre-veraison, veraison, and ripening) in pot-grown plants, while Def and CT were carried out on field-grown plants. Environmental and vegetative parameters were monitored during the experiments. The bulk chemical composition of the fruits was determined in juice by Fourier Transform-Infrared (FT-IR) spectroscopy throughout fruit ripening and at final harvest. The results showed that WD reduced soil water content and leaf water status. CT significantly reduced yield per vine, but increased cluster size. Mid to late WD reduced soluble solids by 1%. CT increased sugar content in juice, while Def decreased sugar accumulation. Total acids were higher in the juice from the field vines. Yet, CT lowered malic and tartaric acids. Def increased tartaric acid. Ammonia and alpha amino nitrogen were higher in the juice from pot-grown vines, while pH was lowered by Def and raised by CT. It is concluded that Solaris has a remarkable ability to tolerate and recover from WD. CT and Def significantly affected the bulk chemical composition of grapes in terms of total acidity and sugar accumulation, with CT grapes having the highest sugar content and the lowest total acidity and Def the opposite.

## 1. Introduction

The continuous emission of greenhouse gases from natural systems and human activities has led to an increase in global temperatures of about 1 °C above the pre-industrial level [1]. Climate change combined with new robust cultivars, well-adapted to short and cool/cold seasons, are main drivers in the development of new viticultural areas in North Europe [2]. In particular, the Scandinavian countries count over 150 wine-producing companies (120 in Denmark, 30 in Sweden, and 12 in Norway) with the grape cultivar Solaris being one of the most grown grapes [3]. In the year 2000, Denmark and Sweden were recognized by the European Union as new regions for commercial wine production, and viticultural development in Denmark has since shown an exponential growth, reaching 170 ha in 2022 [4]. Historically, viticultural areas have been identified on a scale from cool to warm regions [5], with the coolest regions (also known as cool-climate viticultural areas), such as Champagne in France and Rheingau in Germany, having average temperatures of about 14.5 °C (first half of the 20th century) in the growing season (April–October). With a base temperature of 10 °C, this represents a heat sum of approximate 1000–1100 °C. In the same period, the average temperature in Denmark (country average) was approximately 12 °C (±0.3), representing a heat sum of only about 600 (Figure S1). Thus, despite the global temperature increase, Denmark is still cooler than traditional cool climate areas.

Amongst the cultivars associated with the coolest regions were Müller Thurgau, Bacchus, and the Pinot’s [5]-cultivars which would have great difficulties in getting ripe in a climate as cold as the Danish. Oppositely, Solaris is a hardy cultivar of *Vitis vinifera* (L.) that thrives in Denmark. It was created in 1975 at the grape breeding institute in Freiburg (Germany) by Norbert Becker crossing the interspecific grape variety “Merzling” (*V. rupestric* and *V. linsecumii* backcrossed with *V. vinifera* for 6 generations) with a hybrid variety obtained by crossing “Zarya Severa” (*V. amurensis* as a grandparent) and “Muscat Ottonel” (*V. vinifera*). Solaris is an early-ripening grape with good resistance against fungal diseases and to frost [6,7,8]. Due to its good sensory qualities [9,10,11,12] and its adaptation to a short season, it is increasingly grown in northern European countries with marginal climate for winemaking, such as Belgium, the Netherlands, England, Poland, Sweden, and Denmark [3,13]. But it also finds its way to the cooler areas in the Alp region [14,15].

The cultivar grown interacts in complex ways with climate and viticultural practices with strong impact on plant and fruit development, and therefore also on fruit quality [16]. Irrigation and crop thinning have been shown to have a positive impact on both berry weight and berry number per cluster [16]. Amongst viticulture practices, irrigation, especially in more arid regions, is essential to wine production. Water availability may vary considerably from year to year and location and affect the plant differently depending on the phenological stage, yield, and vigor levels. These complex interactions lead to inconsistent and inconclusive results. For this reason, the impact of water deficit on fruit and wine quality is still debatable [17,18,19,20].

**Figure 1:**
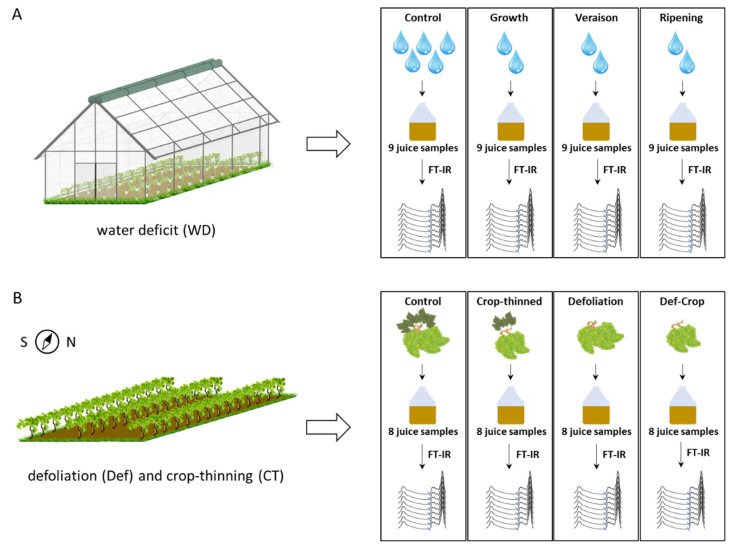
Experimental design. The experiment consisted of two independent trials, one performed in an open screenhouse (**A**) and the other in a conventional field vineyard (**B**) (Schemes S1 and S2). The open screenhouse trial was based on water deficit (WD) studies while the field study looked at the effects of viticultural practices, namely defoliation (Def) and crop reduction (CT). Juice samples (8 per treatment in the vineyard and 9 per treatment in the open screenhouse) were produced from grapes collected from both experiments and then analyzed by Fourier Transform-Infrared Spectroscopy (FT-IR, WineScan, FOSS Analytical A/S, Hillerød, Denmark).

This study is the first published experiment where the impact of different growing conditions and management techniques, such as water deficit (WD), defoliation (Def), and crop-thinning (CT), are investigated in the cool-climate grape variety Solaris grown in the cold viticultural environment of Denmark. An overview of the experimental design is given in Figure 1. The present investigation (Part I) describes alterations in the bulk metabolome during fruit ripening and at harvest as expressed in juice samples measured by Fourier-transform infrared (FT-IR) spectroscopy. In the present study, three main questions are addressed: (1) does WD at different phenological stages affect fruit development and final quality? (2) how do CT and Def change fruit development and their chemical composition? (3) how do these two practices complement each other concerning development and fruit quality/juice composition? This study is followed up by an in-dept metabolomics investigation of mature fruit juice samples and resulting wine samples using proton nuclear magnetic resonance (^1^H NMR) spectroscopy and multivariate data analysis and is published as a separate study (Part II).

## 2. Results

### 2.1. Climate

Experiments were conducted at the Pometum (Taastrup, Denmark), the fruit and berry Genebank, and experimental orchard of the University of Copenhagen. Diurnal and nocturnal temperature fluctuations were monitored throughout the length of the experiments (July and August 2018). The average temperature as measured at the climate station at the University campus (Taastrup, Denmark) was 20.5 and 18.6 °C in July and August, respectively. In the same period, according to DMI (Danish Meteorological Institute), the average national temperature was 19.2 and 17.5 °C, which was 3.6 and 1.8 °C above the 30-year norm used at the time (1961–1990). In the open screenhouse, an average diurnal temperature increase was measured to +1.8 and +0.8 °C in July and August, respectively. On average, temperature was slightly higher in the open screenhouse when compared to the open field, with a diurnal difference of +1.9 °C (from 7 am to 10 pm) and slight difference of +0.2 °C during the night (from 10 pm to 7 am). Overall, 2018 was an unusual warm year where the heat sum (10 °C base), based on the country average daily temperatures, reached 1000 °C for the first time since the meteorological measurements started by DMI in 1874 [21] (Figure S1). In the warmer places like the Pometum, a total heat sum of 1200 °C was reached (Based on temperatures from the University official climate station). In the coolest regions of Denmark (Northern Jutland) a heat sum between 910 °C and 930 °C was reached (www.dmi.dk/vejrarkiv/ accessed 3 March 2022). During the three climate norm periods, from 1874–1960, the annual precipitation was in average 652mm, which in the new norm period 1991–2020 has increased to 759 mm, representing an increase of 17%. The experimental year 2018 was an unusual dry year with only 595 mm [21]. The soil water % was monitored in the field by time-domain reflectometry (TDR) and showed a slow but steady decrease from around 17% in 25 cm depth and 19% in 50 cm in early July, to 14–15% in 25 cm and 16–17% in 50 cm in early August. After 6 days of rain in the period 9–14 August giving a total of 57 mm, soil moisture increased in the field to 24–26% and remained above 20 for the rest of the month (data not shown). The grapevines in the field did not show any signs related to water deficit during the experiment.

### 2.2. Impact of Water Deficit (WD) on the Vegetative Parameters of Vines

The water stress experiment in the open screenhouse was designed to match three phases of berry development, namely after flowering/early fruit development (early stress, 33–54 days after anthesis, DAA), lag-phase to early ripening (mid stress, 55–75 DAA), and ripening (late stress, 76–97 DAA). Each stress period lasted 3 weeks. Harvest was performed at 98 DAA. Vines in the screenhouse were diverse in terms of pruning types and fruiting history and were categorized in 4 groups (Table S1). The stressed groups showed clear drops in soil water content and the typical signs of water deficit, such as yellowing of leaves and decreased leaf water potential (Table 1 and Table 2). Measurements were performed from day 1 in each phase, but it took a few days for the stress levels to be established. Therefore, data shown in Table 1 and Table 2 comprise measurements from day 3. Following WD, soil moisture and leaf water potentials were back to normal levels after about a week. This is reflected in a smaller reduction in water content as an average of the whole phase compared to control. Soil water content was measured twice a day, but no significant difference was observed between the 2 time points. Measurements of leaf water potentials were not initiated before the phase 2 (mid stress period).

Overall, single-and double-cane vines were similarly affected by WD, with differences being clearly shorter primary shoots in double cane vines and higher number of shoots (Table 3). On days with the highest levels of water stress e.g., days with high temperatures and full established treatments, plants with the highest yields tended to show the lowest leaf water potentials (data not shown). Based on the higher number of shoots, total leaf number, total leaf fresh weight, and total leaf area were higher in plants with two fruiting canes (Table 4). Leaf area per shoot was, however, much lower in double-cane vines. Lateral shoot fresh weight was much smaller in stressed single cane plants and slightly smaller in double cane plants, as compared to their respective controls. Both secondary and total leaf area of all shoots were reduced by WD, more severely in single cane plants, with double cane plants showing lower values for secondary leaf area but were higher in total leaf area because of higher primary leaf area (Table 3 and Table 4).

### 2.3. Impact of Water Deficit, Defoliation and Crop-Thinning on Yield and Cluster Weight

An overview of the impact of WD, Def, and CT on fruit yield per plant and average cluster weight is given in Table S2. The average yield was similar (~7 kg per plant) when comparing control vines from field and open screenhouse. Yet, the average cluster weight was higher in control plants from the open screenhouse (~220 g). CT exerted a more negative impact on the yield per vine than Def and WD. In the open screenhouse experiment, no significant effect on yield or cluster size was found (Table S2). As for the field experiment, yield per vine was significantly lowered by crop reduction as it went down from ~7 kg to 1.7 kg (Table S2). The average cluster weight was significantly higher in both CT treatments (~210 g/cluster) compared to control and Def (~170 g per cluster). Overall, CT raised the cluster weight of about 40 g.

### 2.4. Analysis of Juice Samples by FT-IR (WineScan)

#### 2.4.1. Impact of WD as Measured at Harvest

At the final harvest, only minor group-related differences could be observed in the bulk parameters measured on the juice samples from the open screenhouse experiment (Table S3). WD tended to lower the sugar content (glucose + fructose) in the later phases of stress. Malic acid was lower in all treatments, regardless of timing, while tartaric acid tended to be augmented by WD, especially early and late. Parameters for total soluble content (°Brix and density) all showed similar patterns, with the control group and the early stress group showing a tendency to higher values than the later stress treatments. Ammonia and alpha amino nitrogen (αAN) were found to be very similar in all groups. Only the early and mid-stress groups had slightly enhanced levels of ammonia and αAN. Potassium values were also equal among the groups. The pH of grape juice was not influenced by WD at any time.

#### 2.4.2. Impact of WD on Fruit Development

**Figure 2:**
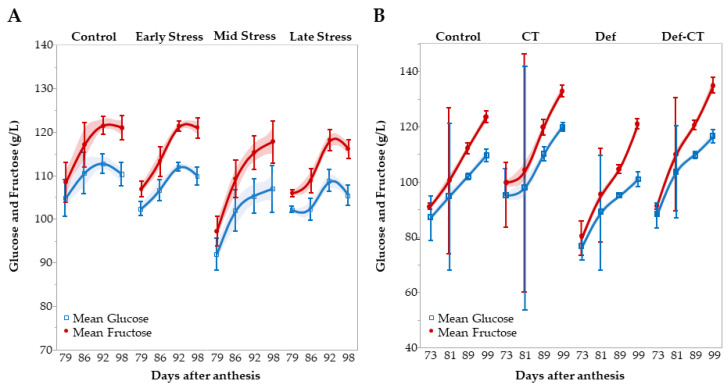
Development during ripening in glucose and fructose in water stress (**A**) and field (**B**) trials. Keys: ‘Control’ = no treatment; ’CT’ = crop-thinning; ‘Def’ = defoliation; ‘Def-CT’ = defoliation and crop-thinning. Vertical bars show the 95% confidence intervals. Shaded areas indicate 95% confidence of fit (spline function).

Inspection of the fruit development during the ripening period (last 3 weeks), from approx. 75 days until harvest 99 DAA, showed a clearer effect of WD. Sugars accumulated significantly faster in the plants which had been stressed in the previous 3 weeks (mid stress) (Figure 2A). Even though the fruits of the stressed plants had lower starting sugar levels, final sugar accumulation in mid-stressed plants had doubled when compared to the control and early stressed plants. The accumulation is even three times higher than the plants being stressed during the late ripening period (late stress) (Figure 2A). More details can be found in Table S4. Fructose is the dominant sugar accumulated accounting for approximate 2/3 of the accumulated sugars during the final weeks. However, the difference among treatments is largest for glucose. Glucose is almost not accumulated in the late-stressed plants (+3.2 g/l ± 1.8), while it accumulates at a 5-fold higher rate in the plants just relived from stress (mid-stressed plants, +15.0 ± 7.5) (Table S4 and Figure 2A).

**Figure 3:**
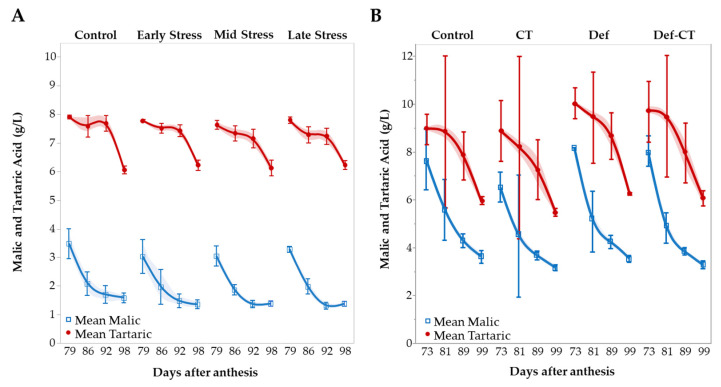
Development during ripening in tartaric and malic acid in water stress (**A**) and field trials (**B**). Keys: ‘Control’ = no treatment; ‘CT’ = crop-thinning; ‘Def’ = defoliation; ‘Def-CT’ = defoliation and crop thinning. Vertical bars indicate 95% confidence intervals. Shaded areas indicate 95% confidence of fit (spline function).

Total acidity decreased to the same extent in all plants (Figure S4A) and the reduction is almost equal for tartaric and malic acid (1.5–1.9 g/L) (Figure 3A and Table S4). However, the decrease in tartaric acid appears to be more a sudden final drop, while malic acid decreases more gradually and earlier. The reduction in acidity is also expressed in a slight increase in pH, regardless of treatment, and is closely (negatively) correlated to the total acid decrease (Figures S6A and S4A, Table S4).

The changes in nitrogen content are dominated by a gradual decrease in ammonia during the ripening period (Figure S5A). αAN decreased during the early weeks but showed a final marked increase, bringing the final harvest level a little higher than at the start of the phase. As for the sugars the increase in αAN is significantly higher in the mid stressed plants compared to the other treatments (Table S4).

#### 2.4.3. Impact of Def and CT as Measured at Harvest

In contrast to what was observed for the open screenhouse experiment, the WineScan analysis of the juice samples from the field experiment (Def and CT) showed several significant group-related differences in the measured parameters (see Table S5). In particular, sugar content and ripeness were significantly impacted by the treatments. More specifically, CT raised levels of glucose and fructose in the fruits, while Def lowered it significantly when compared to the control group. The same pattern is observed in °Brix and density where Def alone led to the lowest measured values, CT to the highest, and the control group to values in between the former two. Total acidity was significantly lower in the juices from the CT plants. The pH was lowered by Def and raised by CT, while tartaric acid was lowered by CT and showed elevated levels by Def. Malic acid was lower in the juice from CT vines, although Def alone did not have a significant impact on its concentration. αAN did not differ significantly among the groups. The juice data from the open screenhouse compared to the open field showed higher levels of amino acids, while malic acid, total acids, and glucose were higher in the juice from the field.

#### 2.4.4. Impact of Def and CT during Fruit Development Relative to WD

The impacts of Def, CT and of different growing conditions (screenhouse vs field) on grape quality are further detailed by including the dynamics of the ripening phase (Figure 2A,B and Figures S4–S6). Grape maturity (as expressed by the sugar content, Figure 2B) generally started at a lower level in the field, especially in the defoliated plants, while the CT plants reached levels similar to the mid-stressed plants (Figure 2A). This illustrates the delay in development caused by the mid stress WD. Final sugar levels are in the CT plants (including Def-CT) ending at a higher level than in any other treatment. Final sugar contents of both fructose and glucose are similar in the control plants across the experiments (Figure 2A,B). The level of ‘physical’ maturity appears to reach a general higher level in the screenhouse as the sugar levels are constant or tend to decrease at the two last sampling dates. Differently, in the field plants a steep increase in sugar levels can be observed also in the final week. The strong inhibitive impact of late stress on sugar accumulation (especially glucose) is magnified when compared to the strong and very equal accumulation achieved in all field treatments (even in Def plants, Figure 2A,B). The development in °Brix supports the above overall picture (Figure S4A,B).

The initial acid and pH levels also reflect a lower level of ripening in the field experiment starting at higher acid levels and a lower pH compared to the screenhouse (Figures S4B and S6B). However, the acid decrease is steep and final levels are approaching the screenhouse levels ending up at approx. 2 g/L higher (see also Tables S3–S5). The steep decrease in acidity observed in the field relate to decreases in both tartaric and malic acid (Figure 3B). The overall pattern mirrors the WD trial with the steepest decrease in tartaric acid in the last week and an earlier (prior to 80–85 DAA) decrease in malic acid (Figure 3A,B). Tartaric acid ends in all cases at approx. 6 g/L while final levels of malic acid are about 2 g/L higher in the field relative to screenhouse plants (Figure 3A,B and Tables S3–S5). Final pH levels remain very low in the defoliated plants (Figure S6B).

Developmental patterns in nitrogen compounds (ammonia and αAN) are relatively similar in field and screenhouse plants. The most striking difference is the different levels of αAN (Figure S5A,B), which in the WD plants was twice as much compared to the field juices.

## 3. Discussion

This study has evaluated the impact of water deficit and viticultural practices on the development of plants and fruits of Solaris vines grown in Denmark. Fruit quality, as expressed in the chemical composition of juice samples made from grapes grown in open screenhouse (WD) and vineyard (Def and CT) conditions, was assessed. The following discussion will address the three fundamental questions constituting the scientific basis of the present investigation: (1) does WD at different phenological stages affect fruit development and final quality? (2) how do CT and Def change fruit development and their chemical composition? (3) how do these two practices complement each other concerning development and fruit quality/juice composition?

### 3.1. Observations on Yield and Vegetative Parameters

The impact on important yield components and quality determinants, including leaf area, cluster size, berry weight/size and total yield, was investigated. Yield is the main parameter used to assess the amount of grape produced per unit surface of vineyard. Even though grape yield is not directly related to wine quality, yield can be used as an indicator to assess the health status of the vines subjected to multiple stress factors. As expected, in the open screenhouse experiment, a higher yield was found in the plants with two fruiting canes compared to one cane, but WD did not influence yield (Table 4 and Table S2). The plant-to-plant yield variation was found to be considerable with a STD of 1.6–1.8 kg/plant. The clusters weight similarly showed great variation masking any possible treatment impact. However, cluster size tended to decrease from control to early- and mid-stressed plants, indicating that WD in the first growth phases (early fruit development and lag phase) may have a negative impact on fruit development. A reduction in fruit set percentage and/or cell division will both have persistent impact. The prevalence of xylem flow both in and out of the berry during these phases may cause WD to mediate significant stress on the berry development compared to the ripening phase [22]. A reduced stomatal conductance may furthermore reduce the photosynthetic supply of carbohydrates.

### 3.2. Observations on Defoliation

Defoliation (Def) did not show a yield-reducing effect. Decrease in yield following Def is often observed in wine production and has been associated with carbon source limitation [23,24,25]. However, this has been observed especially related to strong and early Def [26]. In the present study, Def was performed later and gradually as it was split in two “one-sided” workloads. The first (on the east side) in the end of June (3 weeks after anthesis) and the second (on the west side) 3 weeks later in July. The initial carbon source limitation caused by Def can therefore be expected to be moderated in the present experiment. Furthermore, Solaris is a cultivar high in vigor, with rather big leaves, capable of building a very strong canopy with a high carbon source capacity (Table 3 and Table 4, Schemes S1 and S2).

Def did not change malic acid degradation, but it did decrease sugar accumulation (Table S5, Figure 2B and Figure 3B). This is surprising as Def has often been linked to a lower berry water content and a lower acid content [27,28,29,30]. Especially malic acid is known to show a temperature related degradation during ripening due to higher fruit temperatures caused by the increased sun exposure [31,32]. However, several secondary factors can come into play as a function of Def, and the most likely explanation is that the decrease in total water loss by a reduced canopy made more water available for berry cell expansion. Moreover, a strong influence of the soil-climate-variety-matrix can be assumed especially due to the unusual warm and dry summer of 2018 (Figure S1).

Tartaric acid was found to be higher at harvest in the open screenhouse samples (Figure 3A,B). Previous research has suggested that higher light exposure and temperature can increase tartaric acid synthesis [29,33]. This may also be reflected in the high initial levels during ripening in Def and Def-CT plants (Figure 3B). In the open screenhouse, temperature was found to be slightly elevated during daytime (+1.9 °C) compared to open field. This is not dramatic but probably enough to explain the differences in acid profile including the higher concentrations of malic acid found in the field samples (Figure 3A,B, Tables S3 and S5). Previous research has also shown that it is degraded as a function of increasing temperatures [32], while it increases with increasing irrigation [34].

The content and development of acids are also ripeness indicators, in particular malic acid, which tended to be slightly lowered in all water stress treatments (Table S3). In contrast, Def delayed fruit development resulting in high malic acid levels early in the ripening phase (Figure 3B).

### 3.3. Observations on Crop-Thinning

CT significantly decreased yield, while it increased the average cluster weight and °Brix compared to the control, even in combination with Def (Table S2 and Figure S4B), supporting the picture of a strong source capacity in Solaris. CT normally leads to a higher source-to-sink ratio, being the capacity of the plant to supply carbohydrates divided by the demand for carbohydrates. Usually, the source is characterized by the leaf area, while the sink strength/demand primarily is the sum of the total fruit sink strength, and the vegetative sink strength created by the growing shoot tips [35].

As for the field trial, some groups were designed for favorable ripening conditions by a high source-to-sink ratio. CT increased sugar content to a significantly higher level (Table S5 and Figure 2B) and similar results are reported in literature [27,36,37]. Density and °Brix were higher in the juice samples from CT plants (Table S5 and Figure S4B). Significantly lower sugar and ripeness levels were observed for the Def treatment, which displayed the lowest value measured of °Brix (Table S5 and Figure S4B). The control group showed values in between the Def and the CT levels, illustrating the counteracting effects of the abovementioned practices on sugar content (Table S5). The combined treatment of both Def and CT showed similar sugar levels as the CT treatment alone, implying that the sink reduction was so intense that it outweighed the effect of source reduction (Table S5 and Figures S2 and S3). Even with a severely reduced canopy, sugar levels can be elevated when an intense CT is additionally performed. It is the resulting leaf/fruit balance which counts [38].

### 3.4. Observations on Water Deficit

As for the screenhouse trial, the elevated temperatures and constant water supply in the open screenhouse provided an environment for a higher rate of ripening and better sugar accumulation. However, irrigation was not always carried out for all design groups, which could have photosynthesis-suppressing effects and therefore hinder sugar accumulation. This was clearly demonstrated by the impact of mid and late WD treatments on especially glucose.

The two last stress treatments tended to lower the sugar levels, °Brix and density (Table S3, Figure 2A and Figure S4A). This tendency would imply that WD during the ripening phase can decrease sugar accumulation, which is in agreement with previous studies showing reduced sugar loading as a consequence of water stress [34,35,36]. The mechanisms for suppressing sugar loading are mainly related to the plant’s reaction to water deficit, including closure of stomata to reduce water transpiration [39]. This also leads to a reduced CO_2_ uptake, a decreased carbon assimilation, and therefore lower sugar production. However, the first reaction to decreasing water availability is the termination of leaf and shoot growth [39]. This decrease happens earlier (less negative leaf water potentials) than the reduction in stomatal gas exchange and forms the basis of benefits in partial root-zone drying and deficit irrigation strategies [39,40] leading to an enhanced water use efficiency combined with the benefits of reduced canopy density. The WD treatments resulted in reduced lateral shoot development (Table 4). The strongly enhanced sugar accumulation during the final fruit ripening observed in the mid-stressed plants may be favored by reduced competition from vegetative sinks (Figure 2A). Combined these data documents a strong ability of Solaris to recover from water stress. A similar reduced lateral shoot development was also found after WD in Pinot and Riesling grapes by Reynolds and Naylor [41]. Combined these data documents a strong ability of Solaris to recover from water stress.

### 3.5. Observations on Potassium and Nutrients/Amino Compounds

Parallel to the higher inflow of carbohydrates to the fruits, a higher influx of potassium and water is observed when crop load decreases [27]. It was also observed by elevated levels of potassium in CT (field trial) and a tendency to highest levels in control and lowest in late WD plants (Table 3 and Table 4). Potassium levels were reduced in juices from stressed plants (primarily the late stressed) and enhanced in the CT plants (field trial) (Tables S3 and S5, respectively). Potassium is a very mobile micronutrient, and its uptake, apart from general availability, is regulated by the amount of water influx into the plant. Accordingly, it makes sense that the irrigated control group showed higher values of potassium and the CT treated may be assumed to have experienced a better water balance due to lower fruit load. However, it can be argued that the small variations in potassium values are due to small differences between treatments of samples, e.g., crushing and pressing [10], as well as due to limitations in measuring accuracy by the indirect FT-IR calibration (see material and methods).

The juice samples revealed a clear difference between the two growing conditions, with the open screenhouse samples displaying higher amounts of amino acids (Figure S5A,B and Tables S3 and S5). This can most likely be ascribed to the controlled fertilization process in the open screenhouse experiment where the plants were well supplied with nutrients in the irrigation water. In contrast, vines from the field absorbed nutrients solely from the ground. Since amino acids are an important nitrogen source for yeast during fermentation [28], this may result in quality differences of the final wine.

## 4. Materials and Methods

### 4.1. Experimental Design

The experiment consisted of two independent trials, one performed in an open screenhouse and the other in a conventional field vineyard. The open screenhouse trial was based on water deficit (WD) while the field study looked at the effects of viticultural practices, namely defoliation (Def) and crop reduction (CT). Juice samples (8 per treatment from the vineyard and 9 per treatment from the open screenhouse) were produced from grapes collected from both experiments and then analyzed by Fourier Transform-Infrared Spectroscopy (FT-IR, WineScan). An overview of the experimental design is given in Figure 1. The study was based only on one vintage, which turned out to be unusual warm and dry resulting in good experimental conditions but also in unusual early maturity.

#### 4.1.1. Open Screenhouse Experiment

A total 36 plants of *Vitis vinifera* cv. Solaris were used for the WD experiment. Plants were grown in 30 l plastic pots in an open screenhouse at Pometum, the University of Copenhagen’s agricultural site, located in Taastrup, Denmark. The setup consisted of 3 rows of 14 plants each. Water stress was induced at 3 different phenological stages of plants, namely pre-veraison, veraison, and ripening. Each treatment lasted 3 weeks. Flowering in the greenhouse occurred at the end of May 2018, which puts stress period 1 (2.7–23.7.) in the berry growth phase (stage 1) overlapping into the lag-phase (stage 2). Stress period 2 (24.7–13.8.) can be considered occurring around veraison, again, slightly overlapping into the start of ripening (stage 3). The final stress period 3 (14.8–3.9.) took place during the final stages of ripening. Plants next to the open screenhouse doors were not included in the study, giving a total of 27 (9 × 3) plants. The vines used for the screenhouse trial were all the same age (4 years) but diverse in terms of pruning type and fruiting histories (Table S1). Irrigation was carried out by an automated centralized drip irrigation system with long tubes running through each of the 3 rows. The flow rate and the irrigation timing and duration was controlled electronically by a central console. In this study, irrigation was set to 3 times a day, namely at 9:30 a.m., 03:00 p.m. and 08:00 p.m. with a total flow rate of 2 L/h per dripper. The irrigation time was set to 20 min. This was in the second stress phase increased to 25 min due to warm weather. The water stress groups were irrigated with 2 drippers per plant (reduced to 1 dripper in the second stress phase), while the fully irrigated groups received water from 6 drippers at the same flow rate resulting in a total irrigation of 12 L per day for the irrigated groups, and 4 L per day for the groups experiencing water deficit. In the second stress phase, these figures changed to 15 L per day and 2½ L per day, respectively. Time domain reflectometry (TDR, TRASE, Soil Moisture Equipment Corp., Santa Barbara, CA, USA) was used to determine the soil moisture content in each pot. The measurements were done 9:15 a.m. and 2:45 p.m. shortly before irrigation using probes (25 cm in length) installed in the pots. For the soil used, a soil moisture content of approx. 35% was determined to represent the full capacity in pots irrigated to run-off. Leaf water potential (ψ) was measured twice a week over the course of each stress period. The measurement was carried out around 11:00 a.m. in a conventional pressure chamber (Soil Moisture Equipment Corp., Santa Barbara, CA, USA) on a fully developed leaf from the shaded side of the canopy.

#### 4.1.2. Vineyard Experiments

The second experimental site was a North-South oriented vineyard in Pometum, where 32 field-grown Solaris plants were chosen and divided in 4 groups of different Def and CT treatments. The vines were grown on SO_4_ rootstocks and planted in 2005. As opposed to water stress, which is difficult to control in a field environment, the variables here were different crop levels and the absence or presence of a full canopy in the fruiting zone. A total of 8 vines were subject to each treatment.

Defoliation (Def) was carried out during the early berry growth phase (before the lag phase and consequently before veraison) and consisted of removing all leaves manually from the fruiting area. It was performed split in two one sided workloads. The first (on the east side) in the end of June (3 weeks after anthesis) and the second (on the west side) 3 weeks later in July.

Crop-thinning was carried out manually by removing all existing clusters except 8, all located around the center of the head of the vine. This was done 1 August, 53 days after anthesis (DAA). Conventional vineyard scissors were used. Removed crop load was measured on a per-vine basis. The reduction in crop load was calculated on a percentage basis by adding weight of clusters removed and final harvest weight to obtain total yield per vine and then dividing by weight of clusters removed (Figure S2). Average percentage of crop removal was 67.1% for the group that was defoliated and crop-thinned (Def-CT), and 69% for the group that was only crop-thinned (CT). One control group was established where no treatments were carried out, except the regular vineyard practices (e.g., hedging of the canopy, removal of weeds growing in between vines and spraying sulfur against fungal disease). The yield and cluster size variability was large among individual plants without any CT (control and Def) (Figure S3).

### 4.2. Preparation and Collection of Juice Samples

Fruit ripening was monitored on a weekly basis by collection of subsamples (single berries picked from random positions in clusters) representing 3 plants. The fruit samples were placed in plastic bags and were crushed by hand. Juice samples were collected after 15 min and were analyzed by FT-IR (WineScan) (see Section 4.3).

At final harvest fruits from the open screenhouse (4 September, 98 DAA) and vineyard (16 September, 99 DAA) experiments were manually harvested. On the same day, all plants from each experiment were destemmed, crushed, and set to macerate for 16 h at 3 °C in small 10 L buckets. Every vine was processed separately. After maceration, the individual yields were pressed for 10 min with a maximum pressure of 3 bars using a pneumatic 20 L hydro press (Speidel, Germany). Two samples were taken after pressing, one stored at 3 °C for 2 days and then analyzed by FT-IR (WineScan).

### 4.3. WineScan Analysis

Samples were analyzed by FT-IR spectroscopy using the WineScan instrument (WineScan FT 120, FOSS A/S, Hillerød, Denmark). The WineScan is a Fourier Transform interferometer which use a 37 µm transmission measurement cell with CaF_2_ windows. The scanning range is from 929 to 5011 cm^−1^ and 12 scans are averaged to produce the final spectrum. This gives a measurement time of approximately 30 s [42]. Prior to measurements, samples were centrifuged at 4000 rpm for 5 min. The WineScan instrument provide information on the fundamental molecular vibrations and the spectra have been calibrated to a long list of important wine and grape juice parameters using advanced multivariate regression techniques. In this work the following grape juice parameters are included: glucose, fructose, °Brix, density, pH, total acidity, tartaric acid, malic acid, ammonia, α-amino nitrogen, and potassium.

### 4.4. Statistical Analysis

One way Analysis of Variance and pair-wice comparison of means using Student’s t-tests was performed using SAS JMP^®^ Pro version 16.0, SAS Institute Inc., Cary, NC, USA.

## 5. Conclusions

Overall, the results show that the cool climate variety Solaris has a remarkable resilience to water stress. The timing of water stress only resulted in short time shifts in fruit development only made visible through the FT-IR based monitoring of the process. Due to strong ability to recover from WD, very similar levels of fruit quality components were reached at final harvest. The effect of CT and Def via the changes in leaf/fruit balance has strong but opposite effects on yield and sugar content of the grapes. Malic acid was only influenced to a minor degree by any of the treatments, but lower in the warmer screenhouse compared to the field. Def delayed ripening and sugar accumulation resulting in higher concentrations of tartaric acid. The content of amino acids in the grapes appear mostly to be influenced by nitrogen availability.

## Figures and Tables

**Table 1:** Soil water content (Volumetric %). Average values (±STD) for measurements starting from day 3 are reported. Average of 2 daily measurements performed starting 9.15 a.m. and 2.45 p.m., each time on 9 plants/treatment. Keys: ‘Control’ = no treatment/full irrigation; ‘Early Stress’: WD after flowering/early fruit development; ‘Mid Stress’: WD during lag-phase-early ripening; ‘Late Stress’: WD during ripening. For each stress group, measurements were performed at different phases, namely during the WD period (light blue), as well as during and after recovering from WD (green). ‘-‘: Not measured. Significance is indicated when letters (a, b, c) following values are different within each phase.

	Time Period of Measurement		
Plant Group:	Phase 1	Phase 2	Phase 3
Control	24.1 ± 4.9 ^a^	24.2 ± 5.9 ^a^	27.3 ± 6.3 ^a^
Early stress	9.6 ± 2.9 ^b^	19.9 ± 6.8 ^b^	22.9 ± 7.4 ^b^
Mid stress	-	7.1 ± 3.4 ^c^	23.1 ± 3.9 ^b^
Late stress	-	20.8 ± 7.6 ^b^	9.3 ± 2.9 ^c^

**Table 2:** Leaf water potential (MPa). Average values for measurements starting 3 days after the beginning of the treatments. One measurement per day starting at 11 a.m. Average of 9 plants. (Legends as in Table 1).

	Time Period of Measurement		
Plant Group:	Phase 1	Phase 2	Phase 3
Control	-	−0.68 ± 0.16 ^a^	−0.61 ± 0.07 ^a^
Early stress	-	−0.72 ± 0.15 ^a^	−0.64 ± 0.06 ^a^
Mid stress	-	−1.2 ± 0.19 ^b^	−0.71 ± 0.08 ^a^
Late stress	-	−0.70 ± 0.07 ^a^	−1.03 ± 0.25 ^b^

**Table 3:** Overview of primary vegetative parameters (PVP) as measured in the water deficit groups (WD). Values represent group averages (±STD). Different letters (a, b, c) indicate significant differences between groups. Keys: P = Primary, L = length, N = number, FW = fresh weight, A = area, SC = single cane, DC = double cane.

PVP	Unit	SC, Control	SC, Stress	DC, Control	DC, Stress
P shoot, L	cm/shoot	142 ± 14.8 ^a^	127 ± 13.7 ^a,b^	114 ± 12.9 ^b,c^	101 ± 15.3 ^c^
P shoot, N	#/plant	15 ± 1.0 ^b^	16.5 ± 0.7 ^b^	22.5 ± 2.1 ^a^	25 ± 3.7 ^a^
P shoot, FW	g/plant	1083 ± 63 ^a^	868 ± 7 ^b,c^	945 ± 87 ^b^	821 ± 106 ^c^
P leaves, N	#/plant	219 ± 27.5 ^b^	234 ± 72 ^b^	323 ± 15.7 ^a^	337 ± 30.2 ^a^
P leaves, FW	g/plant	1488 ± 109 ^a^	1455 ± 178 ^a^	1667 ± 134 ^a^	1657 ± 162 ^a^
P leaf, A	m^2^/plant	5.4 ± 0.72 ^b^	5.3 ± 0.99 ^a,b^	6.2 ± 0.31 ^a^	6.3 ± 0.56 ^a^
P leaf, A/shoot	cm^2^/shoot	3573 ± 327 ^a^	3221 ± 461 ^a,b^	2798 ± 395 ^b^	2597 ± 532 ^b^

**Table 4:** Overview of secondary vegetative parameters (SVP) and total plant values including yield, as measured in the water deficit (WD) groups. Values represent group averages (±STD). Different letters (a, b, c) indicate significant differences between groups. Keys: L = lateral, FW = fresh weight, A = area, SC = single cane, DC = double cane.

SVP	Unit	SC, Control	SC, Stress	DC, Control	DC, Stress
L shoot, FW	g/plant	155 ± 76 ^a^	63 ± 14 ^b^	69 ± 22 ^b^	55 ± 30 ^b^
L leaves, FW	g/plant	570 ± 108 ^a^	238 ± 62 ^c^	424 ± 72 ^b^	323 ± 71 ^c^
Total L leaf A	m^2^/plant	2.47 ± 0.50 ^a^	1.22 ± 0.18 ^c^	1.9 ± 0.28 ^b^	1.51 ± 0.35 ^b,c^
Total leaf A	m^2^/plant	7.84 ± 0.54 ^a,b^	6.55 ± 1.17 ^b^	8.13 ± 0.52 ^a^	7.77 ± 0.71 ^a^
A per leaf	cm^2^	245 ± 5.6 ^a^	232 ± 29 ^a^	193 ± 16 ^b^	187 ± 24 ^b^
Leaf A/g fruit	cm^2^/g	13.1 ± 1.6 ^a^	10.6 ± 1.3 ^a^	10.1 ± 2.7 ^a^	10.0 ± 2.4 ^a^
Yield	Kg/plant	6.05 ± 0.96 ^b^	6.18 ± 0.37 ^a,b^	8.33 ± 1.39 ^a^	7.94 ± 1.36 ^a,b^

## Data Availability

The data presented in this study are available on request from the corresponding author. Public data sharing not applicable.

## Supplementary Materials

The following supporting information can be downloaded at: https://www.mdpi.com/article/10.3390/metabo12040363/s1. Scheme S1. Overview of the screenhouse set up with buckets lined up for harvest. The roof and the sides are open and only close in case of rain. Scheme S2. Overview of the field experiment set up with Def-CT plants in the foreground. Figure S1. Development in temperature sums calculated with a 10 °C basis from official 30-year monthly norm values since the start of the measurments by the Danish Meteorological Institute. From the year of the experiments (2018) temperature sums for the country average and the experimental station ‘Pometum’ is shown. Figure S2. Crop reduction in % calculated by adding weight of clusters removed and harvest weight of remaining 8 clusters and dividing by weight of clusters removed. Keys: ‘CT ‘= crop-thinning, ‘Def-CT’ = Defoliation in combination with crop-thinning. Figure S3. Correlation between yield and cluster size. The two crop-reduced treatments ‘CT’ and ‘Def-CT’ were reduced to 8 clusters/plant resulting in a linear correlation of yield to cluster size. ‘Def’ = defoliation. Figure S4. Development of total acidity and °brix during ripening in the water stress (A) and field trials (B). Keys: ‘Control’ = no treatment; ‘CT’= crop-thinning; ‘Def’ = defoliation; ‘Def-CT’ = defoliation and crop thinning. Vertical bars indicate the STD. Different letters indicate significant difference between treatments on a given day. Figure S5. Development of ammonia and alpha amino nitrogen during ripening in the water stress (A) and field (B) trials. Keys: ‘Control’ = no treatment; ‘CT’= crop-thinning; ‘Def’ = defoliation; ‘Def-CT’ = defoliation and crop thinning. Vertical bars indicate the STD. Different letters indicate significant difference between treatments on a given day. Figure S6. Development of pH during ripening in the water stress (A) and field (B) trials. Keys: ‘Control’ = no treatment; ‘CT’= crop-thinning; ‘Def’ = defoliation; ‘Def-CT’ = defoliation and crop thinning. Vertical bars indicate the STD. Different letters indicate significant difference between treatments on a given day. Table S1. Distribution of pruning types and fruiting histories among groups. Table S2. Yield (Kg) and average cluster weight (g) as recorded for plants grown in screenhouse and field. Different letters stand for statistical significance (*p* < 0.001). Table S3. Results from the WineScan analysis of the juice samples from the water stress trial. Keys: ‘Control’ = no treatment; ‘Early stress’ = after flowering; ‘Mid Stress’ = lag phase to early ripening; ‘Late Stress’ = ripening. αAN = Alpha-amino nitrogen. ANOVA was performed to assess the variation between different groups. Different letters stand for statistically significant differences between the groups (*p* < 0.05); ns = not significant. *: calculated as tartaric acid equivalents. Table S4. Change in fruit quality parameters during the late stress period between 16 August and 4 September. (79 to 98 days after anthesis). Keys as before. Table S5. Results from the WineScan analysis of the juice samples from the field trial. Keys: ‘Control’ = no treatment; ‘Def-CT’ = defoliation and crop thinning; ‘Def’ = defoliation only; ‘CT’= crop-thinning. αAN = Alpha-amino nitrogen. ANOVA was performed to assess the variation between different groups. Different letters stand for statistically significant differences between the groups (*p* < 0.05); ns = not significant. ^*^: calculated as tartaric acid equivalents.
