# Supplementary material for: Effects of Water Stress, Defoliation and Crop Thinning on Vitis vinifera L. cv. Solaris: Part I: Plant Responses, Fruit Development and Fruit Quality
Source: Metabolites. 2022 Apr 18;12(4):363. doi: 10.3390/metabo12040363 (PMC9029630; doi:10.3390/metabo12040363)
Supplement: Supplementary file 1 [file metabolites-12-00363-s001.zip › metabolites-1660520-supplementary.pdf]

Supplementary information

Effects of water stress, defoliation and crop thinning on *Vitis vinifera* L. cv. Solaris: Part I: Plant responses, fruit development and fruit quality.

Violetta Aru [1†], Andreas Paul Nittnaus [2,3†], Klavs Martin Sørensen[1], Søren Balling Engelsen[1,] and Torben Bo Toldam-Andersen[2*]

[1] Department of Food Science, University of Copenhagen, Rolighedsvej 26, DK-1958 Frederiksberg, Denmark; violetta@food.ku.dk (V.A.); kms@food.ku.dk (K.M.S.); se@food.ku.dk (S.B.E.)
[2] Department of Plant and Environmental Sciences, University of Copenhagen, Højbakkegård Alle 13, DK-2630 Taastrup, Denmark; a.nittnaus@gmx.at (A.P.N.); tbta@plen.ku.dk (T.B.T.A.)
[3] Instituto Superior de Agronomia, Universidade de Lisboa, Tapada da Ajuda, 1349-017 Lisboa, Portugal; (A.P.N.)
[*] Correspondence: tbta@plen.ku.dk (T.B.T.A.)
† Equally contributed to this work.

Experimental design.

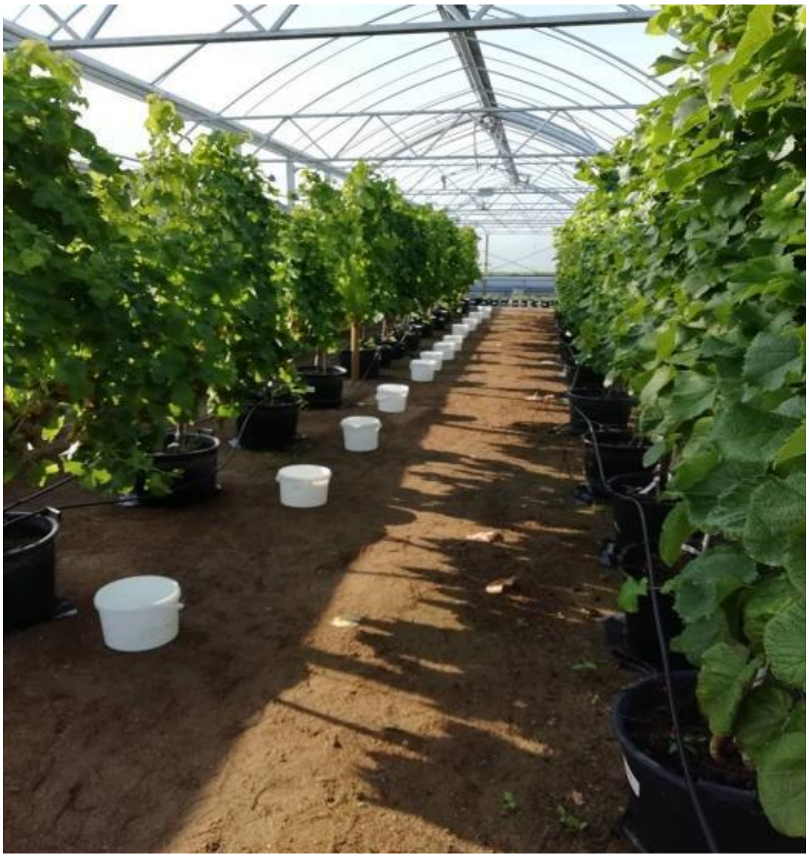

Scheme S1. Overview of the screenhouse set up with buckets lined up for harvest. The roof and the sides are open and only close in case of rain.

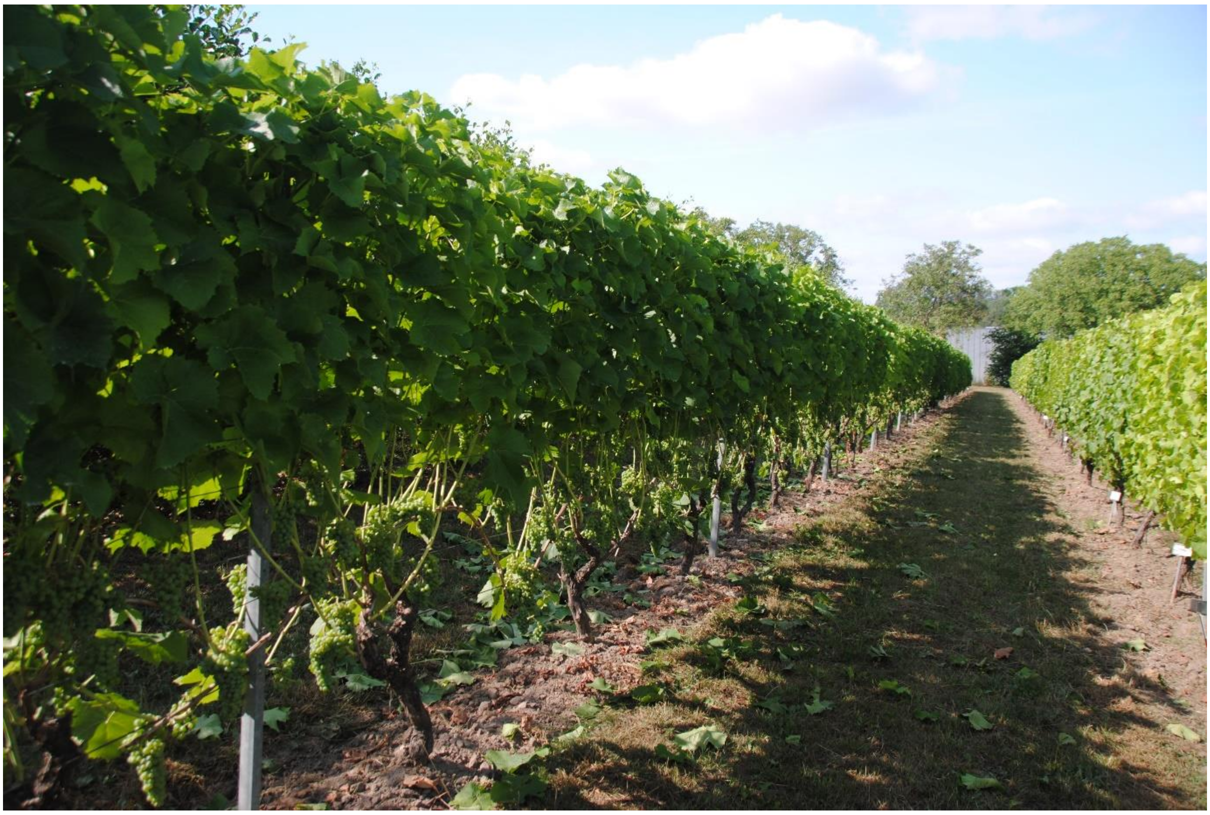

Scheme S2. Overview of the field experiment set up with Def-CT plants in the foreground.

Table S1. Distribution of pruning types and fruiting histories among groups.

	Control	Early Stress	Mid Stress	Late Stress
**Single cane, 1st fruit**	2	2	2	2
**Single cane, 2nd fruit**	3	3	3	3
**Single cane, 3rd fruit**	2	2	2	2
**Double cane, 2nd fruit**	2	2	2	2
**Plants per group**	9	9	9	9
**Plants per row**	3	3	3	3

Results

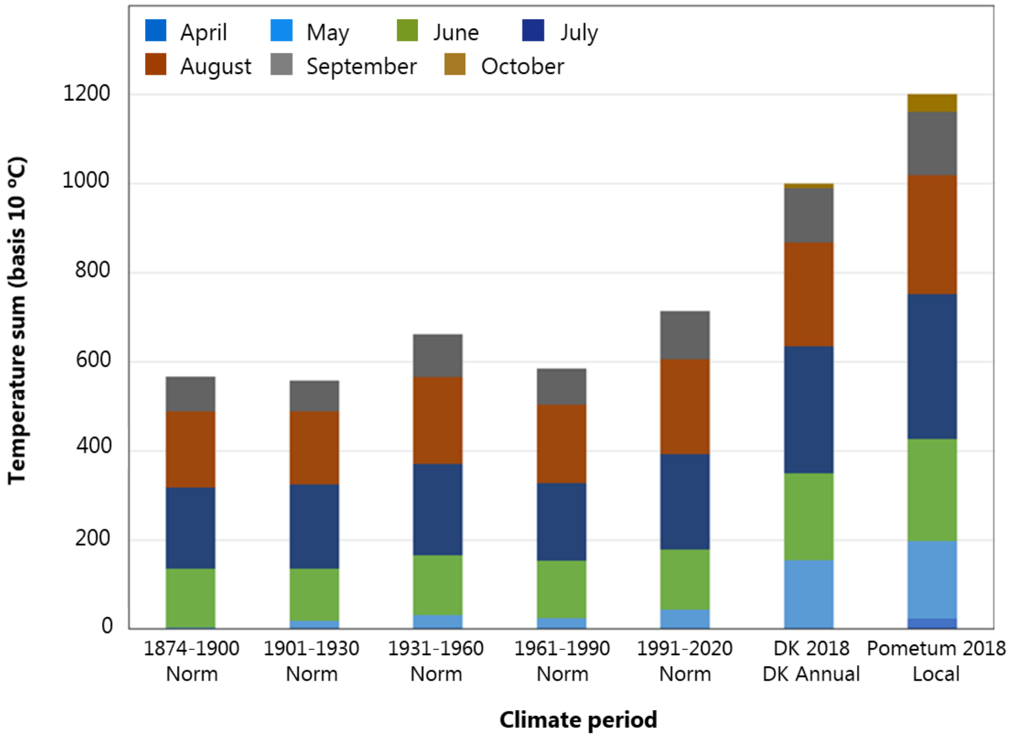

Figure S1. Development in temperature sums calculated with a 10 °C basis from official 30-year monthly norm values since the start of the measurements by the Danish Meteorological Institute. From the year of the experiments (2018) temperature sums for the country average and the experimental station 'Pometum' is shown.

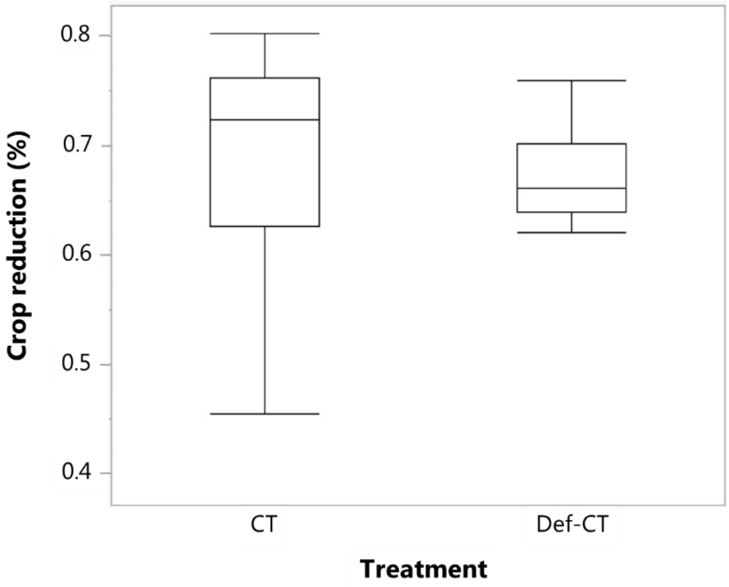

Figure S2. Crop reduction in % calculated by adding weight of clusters removed and harvest weight of remaining 8 clusters and dividing by weight of clusters removed. Keys: 'CT' = crop-thinning, 'Def-CT' = Defoliation in combination with crop-thinning.

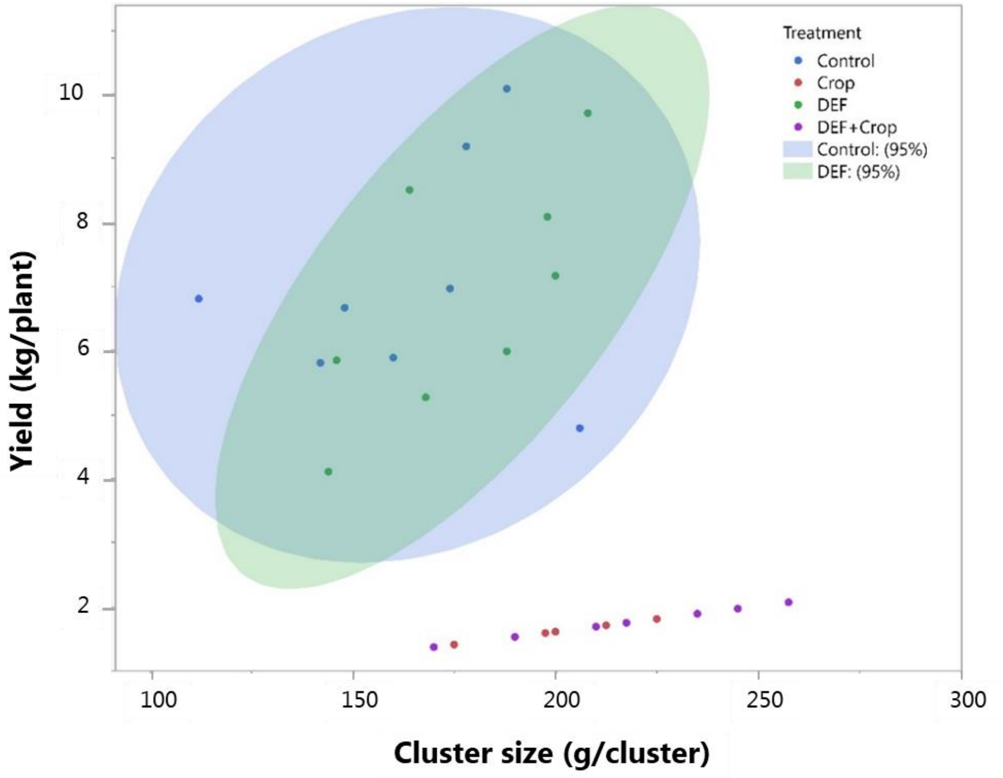

Figure S3. Correlation between yield and cluster size. The two crop-reduced treatments ‘CT’ and ‘Def-CT’ were reduced to 8 clusters/plant resulting in a linear correlation of yield to cluster size. ‘Def’ = defoliation.

Table S2. Yield (kg) and average cluster weight (g) as recorded for plants grown in screenhouse and field. Different letters stand for statistical significance ($p<0.001$).

	Yield per plant (kg)	Average cluster weight (g)
	**Open Screenhouse**	
Control	7.2 ± 1.6^{a}	207 ± 28^{a}
Early stress	7.2 ± 1.6^{a}	188 ± 36^{a}
Mid stress	7.3 ± 1.8^{a}	184 ± 27^{a}
Late stress	8.0 ± 1.8^{a}	194 ± 43^{a}
	**Field**	
Control	7 ± 1.8^{a}	164 ± 30^{b}
Def	6.8 ± 1.9^{a}	177 ± 25^{b}
Crop	1.7 ± 0.2^{b}	207 ± 20^{a}
DefCrop	1.7 ± 0.3^{b}	212 ± 33^{a}

Table S3. Results from the WineScan analysis of the juice samples from the water stress trial. Keys: 'Control' = no treatment; 'Early stress' = after flowering; 'Mid Stress' = lag phase to early ripening; 'Late Stress' = ripening. αAN = Alpha-amino nitrogen. ANOVA was performed to assess the variation between different groups. Different letters stand for statistically significant differences between the groups (p<0.05); ns = not significant. *: calculated as tartaric acid.

Parameter	Control	Early stress	Mid stress	Late stress	*p-val*
**Glu + Fru, g/l**	224 ± 13.5[a]	224 ± 10.8[a]	218 ± 20.4[ab]	215 ± 16.1[b]	< 0.05
**°Brix**	21.7 ±1.13[a]	21.7 ±0.91[a]	21.2 ±1.70[ab]	20.9 ±1.37[b]	< 0.05
**Density, g/ml**	1.094 ±0.006	1.096 ±0.004	1.092 ±0.008	1.091 ±0.006	ns
**Total acidity, g/l***	6.9 ±0.7	6.8 ±0.4	6.7 ±0.5	6.8 ±0.4	ns
**pH**	3.22 ±0.07	3.20 ±0.04	3.19 ±0.06	3.19 ±0.06	ns
**Tartaric acid, g/l**	6.06 ±0.36	6.2 ±0.26	6.1 ±0.38	6.2 ±0.27	ns
**Malic acid, g/l**	1.6 ±0.33[a]	1.4 ±0.25[b]	1.4 ±0.30[b]	1.4 ±0.30[b]	< 0.05
**Ammonia, mg/l**	104 ±16.5[b]	112 ±9.0[a]	113 ±11.4[a]	109 ±10.9[ab]	< 0.05
**αAN, mg/l**	289 ±17[b]	306 ±22[a]	299 ±22[a]	289 ±20[b]	< 0.05
**Potassium, mg/l**	1315 ±211[a]	1241 ±187[ab]	1230 ±193[ab]	1168 ±187[b]	< 0.05

Table S4. Change in fruit quality parameters during the late stress period between 16 August and 4 September. (79 to 98 days after anthesis). Keys as before.

Parameter	Control	Early stress	Mid stress	Late stress	*p-val*
**Glu + Fru, g/l**	13.5 ±5.5[b]	17.2 ±2.3[b]	31.6 ±13.2[a]	9.4 ±3.3[b]	<0.05
**Glucose**	5.5 ±2.9[b]	7.5 ±0.9[b]	15.0 ±7.5[a]	3.2 ±1.8[b]	<0.05
**Fructose**	12.5 ±2.5[b]	14.1 ±0.7[b]	20.6 ±6.6[a]	10.1 ±1.5[b]	<0.05
**°Brix**	1.2 ± 0.5[b]	1.6 ±0.2[b]	2.9 ±1.2[a]	1.0 ±0.3[b]	<0.05
**Total acidity, g/l***	-3.6 ± 0.7	-3.1 ±0.5	-3.2 ±0.5	-3.5 ± 0.2	ns
**pH**	0.23 ±0.06	0.19 ± 0.03	0.20 ±0.04	0.20 ±0.02	ns
**Tartaric acid, g/l**	-1.8 ± 0.1[b]	-1.6 ±0.2[a]	-1.5 ±0.3[a]	-1.6 ±0.1	<0.05
**Malic acid, g/l**	-1.9 ±0.5	-1.7 ± 0.4	-1.7 ±0.3	-1.9 ±0.2	ns
**Ammonia, mg/l**	-29 ±5	-33 ± 8	-31 ±8	-24 ±6	ns
**αAN, mg/l**	6 ±8[b]	7 ± 16[b]	22 ±11[a]	5 ±7[b]	<0.05
**Potassium, mg/l**	153 ±55	166 ±106	315 ±115	128 ±55	0.0059

Table S5. Results from the WineScan analysis of the juice samples from the field trial. Keys: ‘Control’ = no treatment; ‘Def-CT’ = defoliation and crop thinning; ‘Def’ = defoliation only; ‘CT’= crop-thinning. αAN = Alpha-amino nitrogen. ANOVA was performed to assess the variation between different groups. Different letters stand for statistically significant differences between the groups ($p<0.05$); ns = not significant. *: calculated as tartaric acid.

**Parameter**	**Control**	**Def**	**CT**	**Def-CT**	***p-val***
**Glu + Fru, g/l**	220± 7.9^{c}	203± 9.6^{d}	239±6.6^{a}	232 ± 7.3^{b}	<0.05
**°Brix**	21.5±0.7^{c}	20±0.9^{d}	23.2±0.6^{a}	22.6± 0.6^{b}	<0.05
**Density, g/ml**	1.093±0.0038^{b}	1.084±0.0084^{c}	1.101±0.0028^{a}	1.097±0.0035^{a}	<0.05
**Total acidity, g/l***	9.42±0.30^{b}	9.77±0.29^{a}	8.65±0.29^{c}	9.56±0.40^{ab}	<0.05
**pH**	3.10±0.07^{b}	3.00±0.01^{c}	3.14±0.05^{a}	3.08±0.065^{b}	<0.05
**Tartaric acid, g/l**	5.9±0.16^{a}	6.3±0.05^{a}	5.5±0.3^{b}	6.1±0.4^{a}	<0.05
**Malic acid, g/l**	3.6±0.26^{a}	3.6±0.14^{a}	3.2±0.23^{b}	3.3±0.2^{b}	<0.05
**Ammonia, mg/l**	97±18	100±6	98±16	95±2	ns
**αAN, mg/l**	156±24	154±10	150±15	156±11	ns
**FolinC, GAE mg/l**	184±38^{b}	406±149^{a}	237±85^{b}	396±143^{a}	<0.05
**Potassium, mg/l**	950±100^{b}	850±53b	1342±146a	902±69b	<0.05

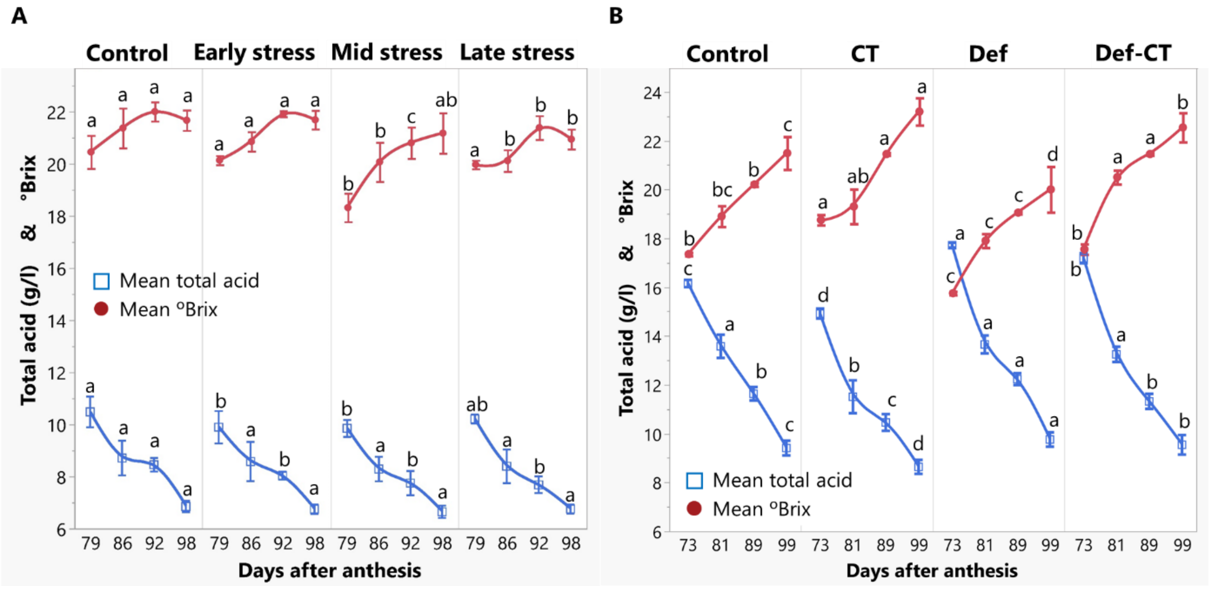

Figure S4. Development of total acidity and °brix during ripening in the water stress (A) and field trials (B). Keys: ‘Control’ = no treatment; ‘CT’= crop-thinning; ‘Def’ = defoliation; ‘Def-CT’ = defoliation and crop thinning. Vertical bars indicate the STD. Different letters indicate significant difference between treatments on a given day.

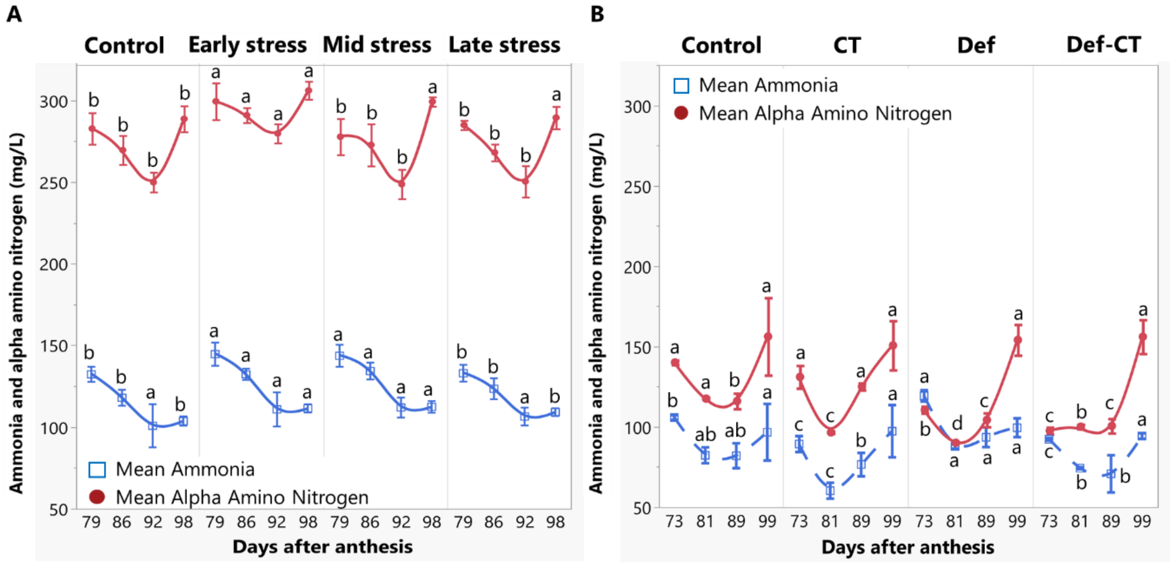

Figure S5. Development of ammonia and alpha amino nitrogen during ripening in the water stress (A) and field (B) trials. Keys: 'Control' = no treatment; 'CT'= crop-thinning; 'Def' = defoliation; 'Def-CT' = defoliation and crop thinning. Vertical bars indicate the STD. Different letters indicate significant difference between treatments on a given day.

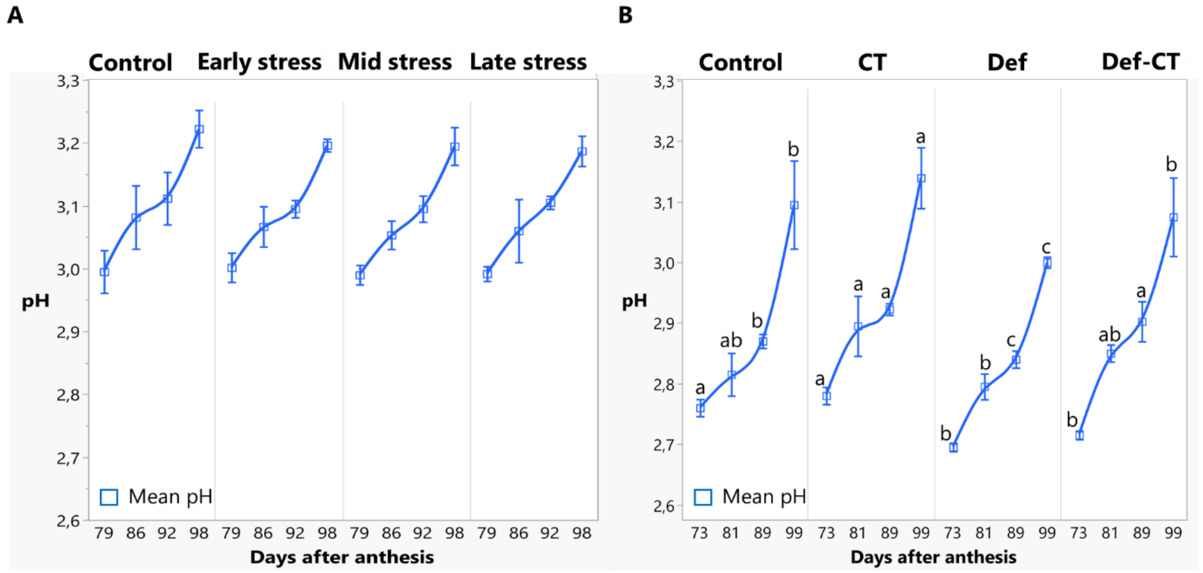

Figure S6. Development of pH during ripening in the water stress (A) and field (B) trials. Keys: 'Control' = no treatment; 'CT'= crop-thinning; 'Def' = defoliation; 'Def-CT' = defoliation and crop thinning. Vertical bars indicate the STD. Different letters indicate significant difference between treatments on a given day.
